# Confusion, knock-down and kill of *Aedes aegypti* using metofluthrin in domestic settings: a powerful tool to prevent dengue transmission?

**DOI:** 10.1186/1756-3305-6-262

**Published:** 2013-09-11

**Authors:** Scott A Ritchie, Gregor J Devine

**Affiliations:** 1 School of Public Health and Tropical Medicine and Rehabilitative Sciences, James Cook University, Cairns, Queensland 4870, Australia; 2 Mosquito Control Laboratory, QIMR-Berghofer Institute of Medical Research, Brisbane 4006, Queensland, Australia

**Keywords:** Dengue, *Aedes aegypti*, Metofluthrin, Synthetic pyrethroid, Control

## Abstract

**Background:**

Dengue control methods are reliant upon control of the vector, primarily *Aedes aegypti*. Current adulticiding methods in North Queensland include treating premises with residual synthetic pyrethroid insecticides (interior residual spraying; IRS), a laborious, intrusive task. The vapor active synthetic pyrethroid metofluthrin might offer an efficient alternative as some studies indicate that it prevents biting and has strong knockdown effects. However, its expellant and/or irritant effects, longevity, residual activity and the speed with which biting behavior is disrupted have not yet been characterized.

**Methods:**

We exposed cohorts of Cairns colony (F2-4) *Ae. aegypti* to rooms (17–24 m^3^) treated with 5% and 10% AI metofluthrin emanators. Using free-flying and caged populations we measured biting (human landing rate), expulsion through unscreened windows, knockdown and death over periods ranging between a few minutes and 24 hrs. Observations of the behavior of single female *Ae. aegypti* exposed to metofluthrin were also made.

**Results:**

Female *Ae. aegypti* exposed to 5% or 10% metofluthrin formulations were almost entirely inhibited from biting. This was the result of rapid knockdown and mortality (80-90% in less than one hour) and to the behavioral impacts of exposure that, within minutes, caused female *Ae. aegypti* to become disoriented, stop landing on hosts, and seek resting sites. Exposed mosquitoes did not exhibit any increased propensity to exit treated rooms and the 10% AI resin remained fully active for at least 20 days.

**Conclusion:**

The new, high-dose, resin formulations of metofluthrin act quickly to prevent biting and to knockdown and kill free-flying female *Ae. aegypti* in our experimental rooms. There was no evidence that metofluthrin induced escape from treated areas. Resin-based metofluthrin emanators show great potential as a replacement for labor intensive IRS for dengue vector control.

## Background

The volatile synthetic pyrethroid, metofluthrin, has great potential for urban dengue control by preventing or reducing contact between humans and the major urban vector, *Aedes aegypti*. Metofluthrin-impregnated paper strips reduced human landings by >90% in large experimental wind-tunnels [1] and indoor resting densities by ca. 50% for several weeks [2]. Rapley *et al.*[3] showed that similar emanators prevented all bites in treated rooms.

Although generally described as a repellent or spatial repellent, with an overwhelming emphasis on measures of mosquito densities within houses or reductions in human landings [1,2,4-8], metofluthrin is also an extremely effective knockdown (KD) and killing agent when applied at sufficient dose. Matsuo [9] describes a range of KD effects (and therefore presumably lethal impacts) for a variety of mosquito species and metofluthrin formulations. Using paper emanators, an exposure of 24 hours was highly lethal to free-flying *Ae. aegypti* released in the confined space of a typical house in northern Queensland [3]. In open house structures in Indonesia, a similar formulation caused 100% mortality among caged *Anopheles balabacensis* >2 meters from the metofluthrin source [6]. Rapley *et al.*[3] found that, under their test conditions, metofluthrin was not repellent or expellant: host-seeking mosquitoes frequently entered metofluthrin-treated spaces as part of their disrupted flight activity. Mosquitoes entering treated rooms rapidly became disoriented, seemed unable to coordinate human landings, sought harborage and were subsequently knocked-down and killed. However, the experimental rooms were screened, preventing mosquito escape.

The potential impacts of metofluthrin on reducing human biting by free-flying mosquitoes, whether as a result of KD activity or behavior require further characterization. This will help assess its potential for breaking disease transmission, and create optimal methods of deployment that result in long-lasting reductions in human-mosquito contact. In particular we need to know how long formulations remain effective in real-world settings, the rapidity with which they prevent mosquitoes from biting, and whether exposed female *Ae. aegypti* display a tendency to exit affected rooms through open windows and doors. If the latter occurs, it is possible that those mosquitoes might pose a transmission risk to nearby, untreated targets [10-12].

This paper describes the response of caged and free-flying female *Ae. aegypti* to high doses of metofluthrin incorporated into polyethylene emanators in domestic settings (SumiOne™ net). We used both 5% and 10% w/w formulations as both were being considered for commercialization by the manufacturer. We measured KD, mortality and expellency, assessed the longevity of the emanators and described the behaviors of affected mosquitoes.

## Methods

Ethics approval for human blood feeding and release of mosquitoes was provided by James Cook Un. H4907; mosquitoes were released with consent of homeowner.

### Metofluthrin and mosquitoes

Metofluthrin emanators (SumiOne™ net), under development by Sumitomo Chemical Corporation, were supplied by Sumitomo Chemical Australia Pty Ltd. We tested 5% or 10% AI w/w formulations in which the chemical is incorporated into a doubled-over polyethylene mesh 8 cm × 15 cm (240 cm^2^ in total). This is equivalent to 106 or 212 mg a.i. / emanator. Unless otherwise specified, the mosquitoes used in our tests were sugar-fed (i.e. blood-starved) *Ae. aegypti* Cairns strain, F3-5, 3–10 days old.

### Behavior, speed of impact, and residual effects

We tested the impacts of metofluthrin exposure by releasing cohorts of *Ae. aegypti* into two experimental rooms (Room 1 = 24.3 m^3^, Room 2 = 22.3 m^3^) in two Cairns households. Both contained typical harbourage areas: dark suitcases, laundry hampers, hanging clothes, storage boxes and had white tiled floors that facilitated the collection of KD and dead mosquitoes. A small table fan (Room 1) or a ceiling fan (Room 2) were set on a low speed to help disperse the molecule and create airflow through the room. Fans are commonly used in North Queensland. The rooms were sealed other than for window exit traps. In Room 1, screened window traps similar to those used by Achee *et al.*[4], covering an area of 0.86 m^2^, were fitted with a sticky panel to capture escaping mosquitoes. In Room 2, mosquitoes were collected from a window trap 1.6 m^2^ in area by aspirator alone. Weather data were obtained from the Bureau of Meteorology station at Cairns airport 2 km north of the trial house; data logger recordings indicated that temperature within the rooms was within 1°C of ambient.

Room 1 was used to assess the effects of a 5% emanator on landing counts, expellency, KD and mortality. Trials were conducted in winter (June) and summer (January). A cohort of 30 female *Ae. aegypti* were released. After a 5 minute acclimation period, a 1 minute human bait count was made to confirm that the mosquitoes were motivated to blood feed and to provide a baseline for comparison post-treatment. The metofluthrin device was then placed 0.8 m above the ground and opened and the observer left the room. One hour later, another 1 minute biting count was made and the observer left the room once more. Preliminary work showed that further observations on landings were unnecessary as almost all mosquitoes were affected (did not bite) within the hour. After 24 hrs, collections from window traps were made.

Mosquito KD and mortality in Room 1 were assessed (1 h and 24 h post treatment) by exposing 10 female *Ae. aegypti* within a Clarke bioassay ring placed 2 m from the device. Experiments were repeated 5 times using single, newly opened emanators.

Room 2 was used to assess the effects of a 10% formulation with the express purpose of demonstrating the rapidity with which human landings might be disrupted. This higher dose was also used to prove that emanators have no discernable residual impact once they have been removed from a treated space. Cohorts of 15 acclimated female mosquitoes were released into a room by a seated observer. Landing counts were made over two minute intervals until 10 minutes had elapsed. Preliminary observations showed that by this time, almost all human landing had ceased. At 20 minutes, all mosquitoes were collected by sweep net or forceps and scored as knocked down, dead or alive. Their position (within the room or in the window trap) was noted. We assumed that insects entering the trap did not re-emerge and, although impossible to deny categorically, the seated observer did not observe mosquitoes reentering the room. Tests were repeated on 8 occasions divided among three separate dates. On each date, a different emanator, open for 14–20 days, was used (bioassay data showed that emanators were similarly active over this period; see Results).

In all cases, each test was separated by a period when the metofluthrin source was removed and the room was “force ventilated” for ≥1 hour (all doors and windows opened and fans set to high). Little mortality was observed in mosquitoes housed with a Clark bioassay ring set in the ventilated room after 1 hr. After that time, the doors were closed, the window openings were returned to their standard configuration and the landing rates of a new cohort of mosquitoes were tested to determine that there were no discernable residues in the room.

### Studies on the longevity of 10% metofluthrin emanators

Room 2 was used to assess the KD and mortality effects of a 10% metofluthrin formulation over time. Two 10% emanators were opened one day apart, and left hanging in an airy room (March 2013; mean temperature 27.1°C) under standard conditions (open windows, ceiling fan set on low, estimated wind speed of 0.5-1.5 m/s). This meant that there were two days on which single emanators, aged for 11, 20 and 30 days, could be tested. These emanators were then suspended 0.8 m from the floor, and 2 m from cohorts of 15 female mosquitoes were confined to bioassay chambers (Clark’s rings) also suspended 0.8 m above the ground. Counts of mortality and KD (unable to fly or walk in a coordinated manner) were made at 0, 5, 10, 20 and 30 minutes. Each treatment was replicated six times over six separate dates in March 2013, room temperatures ranging from 25-29°C. Between replicates the room was “force ventilated” as above.

### Open room studies: knockdown and escape

**Figure 1:**
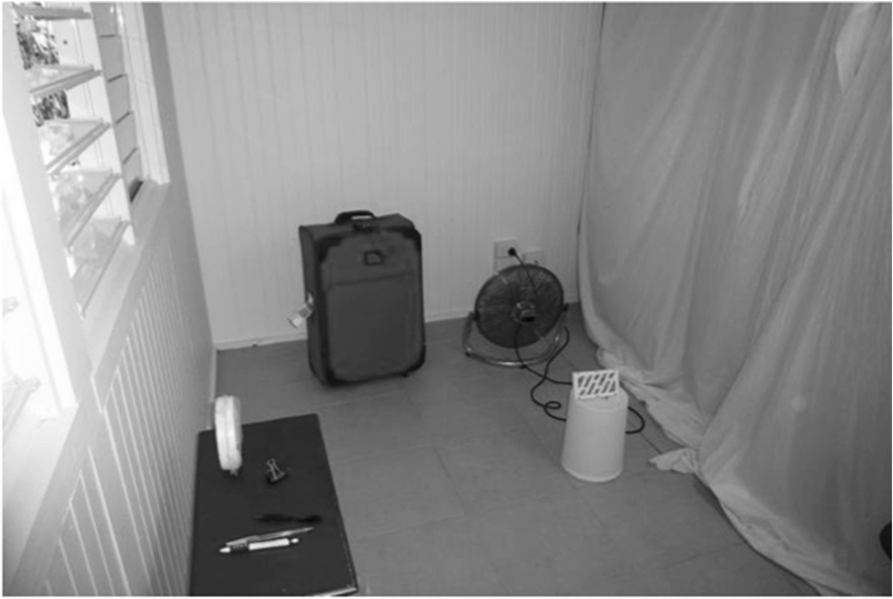
**Experimental room 3.** Room used in open room trials showing a suitcase, metofluthrin emanator (on bucket), open louvered window and Clark sentinel ring on table. The small table fan was turned on low and faced away from the emanator, generating wind speed of 0.2 m/s at the emanator located 1 m way.

The relatively small openings into the window traps used in Room 1 and 2 might inhibit the exit of mosquitoes from the room. In order to allow a simpler escape we used white sheets to adapt another room (Room 3; Figure 1) and create a small experimental space (17.4 m^3^) with two open louvered windows (each 0.54 m^2^). A small table fan was set on the ground, facing away, 1 m from the emanator to disperse the molecule (Figure 1). Two closed black suitcases (24 × 40 × 56 cm) were set on either side of the room to provide harborage for resting mosquitoes. The fan was run at low speed (an airflow of 0.2 m/s at the emanator; Kestrel 1000® wind meter). All trials were conducted for 2 hours during November and December 2011. Temperatures ranged from 23°C to 32°C.

Three treatments of a control (no emanator), a single 5% emanator and a ‘double’ 5% emanator were compared. The latter treatment used two 5% AI emanators opened simultaneously and sandwiched together to approximate the 10% AI emanator (the 10% AI formulation was not available at that time). In each experiment a cohort of 30 female *Ae. aegypti* were released, allowed to disperse and attempt to bite a human volunteer for 2–5 minutes. The emanator was then deployed and the human volunteer left the room for 2 hours. A sentinel bioassay ring containing 5 female *Ae. aegypti* was set in the room to confirm the efficacy of the emanator for each trial. After 2 h, free flying mosquitoes were collected with a sweep net and knocked down mosquitoes were collected from the floor and harborage areas with forceps. Between replicates the room was “force ventilated” as above. For the treatment with two 5% emanators we repeated these experiments using 3–10 day old cohorts of males, starved nulliparous and recently bloodfed females. We also tested cohorts of 7–12 day old gravid females and parous females. Each test was replicated 5 times using emanators that has been open from 0–5 days.

### Behavior of individual, exposed *Ae. aegypti*

The behavior of single 3–10 day old nulliparous female *Ae. aegypti* was observed following exposure to the double emanator (two 5% devices sandwiched together). This was also done in Room 3 with open windows. A single observer recorded the dominant behaviors of biting, flying, resting and KD every 10 seconds. Thus for each minute the total behavioral score = 6. Observations began as soon as biting was attempted and, at that time, the emanators were also opened. Observations continued until the individual mosquito exited through the windows or was permanently knocked down. The observations were repeated 15 times using a new mosquito for each replicate. We then calculated the proportion of behaviors for each minute from the 15 replicates. Between replicates, metofluthrin sources were removed and rooms were “force ventilated”. As a control, we observed the behaviors of single female *Ae. aegypti* over 15 min. in the absence of metofluthrin (n = 10). More detailed, descriptive observations were verbally recorded on an iPhone for 5 of the treated replicates. Trials were conducted between December 2011 and January 2012 with temperatures ranging from 26-32°C.

### Data analyses

The bioassays examining kill and KD by emanators of differing ages were analysed using ArcSin transformed proportional data and repeated measures ANOVA. The graphical presentations are of the back-transformed data. Landing counts and numbers of mosquitoes in the free-flying trials were compared by ANOVA and Tukey’s HSD test.

## Results

### Speed of impact, and residual effects

In the presence of a human and the absence of metofluthrin, cohorts of newly released female *Ae. aegypti* began to land and attempt to bite almost immediately. The number of landings within 1 minute that resulted from releases of 30 females ranged from 20 in the winter to 60 in the summer (Table 1). Mean daily low and high temperatures during these periods were 13.7/25.3°C and 22.8/30.8°C, respectively. Subsequent exposure to 5% metofluthrin rapidly affected all free-flying mosquitoes. After 1 hour, the mean human landing count was zero with 80-90% KD. Complete KD and kill was achieved within 22 hours (Table 1).

**Table 1:** **Impact of metofluthrin (5% AI) on biting and KD of *Ae. aegypti *in winter and summer**

	**1 hour before treatment**	**1 hr post treat**	**22 hour post treat KD**
Winter	32.2 (22.5-42.1)a	0 (0–0)b	ND
(landing rate)			
Winter (KD)	0 (0–0)a	8.7 (8.0-9.4)b	10 (10–10)b
Summer	46.0 (28.6 – 63.4)a	0 (0–0)b	ND
(landing rate)			
Summer (KD)	0 (0–0)a	8.2 (6.9-9.5)b	10 (10–10)b

Mean (CI) human landing rate is a 1 minute human biting count of 30 female *Ae. aegypti* 5 min. before and 1 hr after exposure to a 5% AI emanator. Mean (CI) KD/kill assessed from a cohort of 10 3–10 day old female *Ae. aegypti* held in a Clarke bioassay ring placed 2 m from an emanator. Numbers following CIs in same row indicate significant difference in mean KD number between treatment and control using a t-test (landing rate) or ANOVA (KD), with means separated by Tukey’s multiple comparison test.

**Figure 2:**
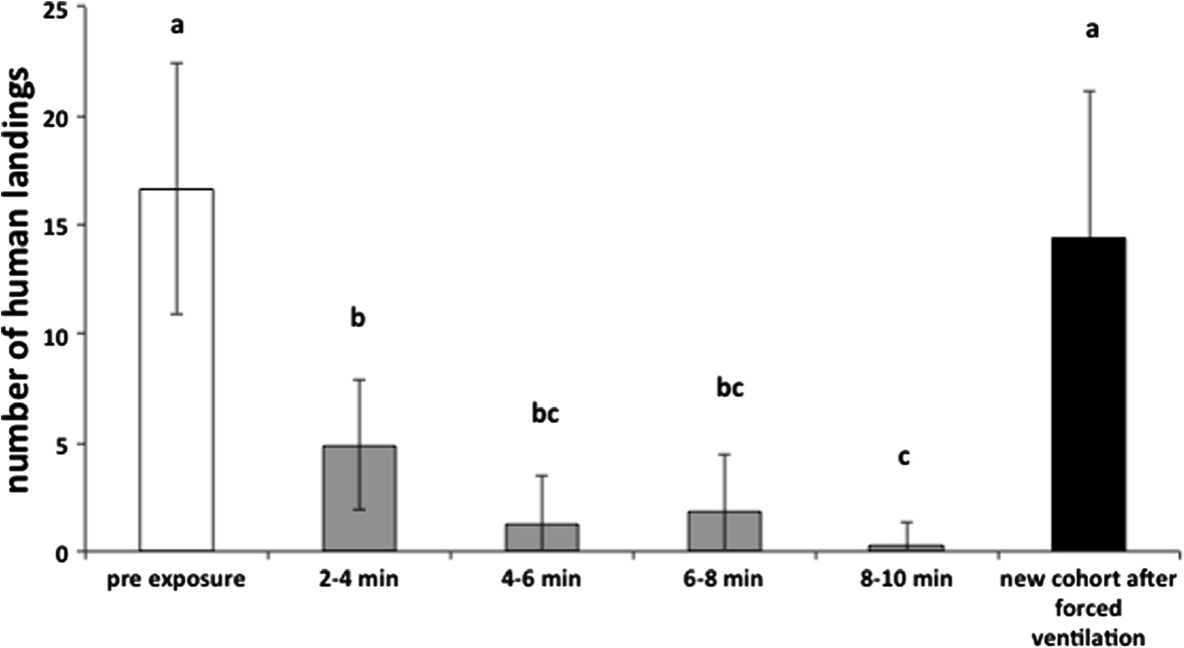
**Mean (CI) human landing rate of female *Ae. aegypti *released into an untreated room then subsequently exposed to a 10% metofluthrin emanator.** Letters above columns depict significant difference using Tukey’s HSD test.

The 10% formulation also affected mosquitoes very rapidly (Figure 2). In the absence of metofluthrin, releases of 15 females led to 17 landings per 2 minute interval. Immediately following exposure this decreased to 5 landings. By 8 minutes, the number of landings was negligible. These reductions were highly significant (ANOVA, F = 55.9, P < 0.0001) and 90% of mosquitoes died within 20 minutes of exposure (Figure 2).

After removal of the 10% metofluthrin source and a 2 h “forced ventilation” of the room, there were no discernable residual effects on landing rates in Room 2. New cohorts of mosquitoes observed after “forced ventilation” displayed 14 landings per two minute period. This was not significantly different to the pre-treatment landing count (Tukey’s HSD test, p = 0.56).

### Escape of exposed *Ae. aegypti* into window exit traps

**Figure 3:**
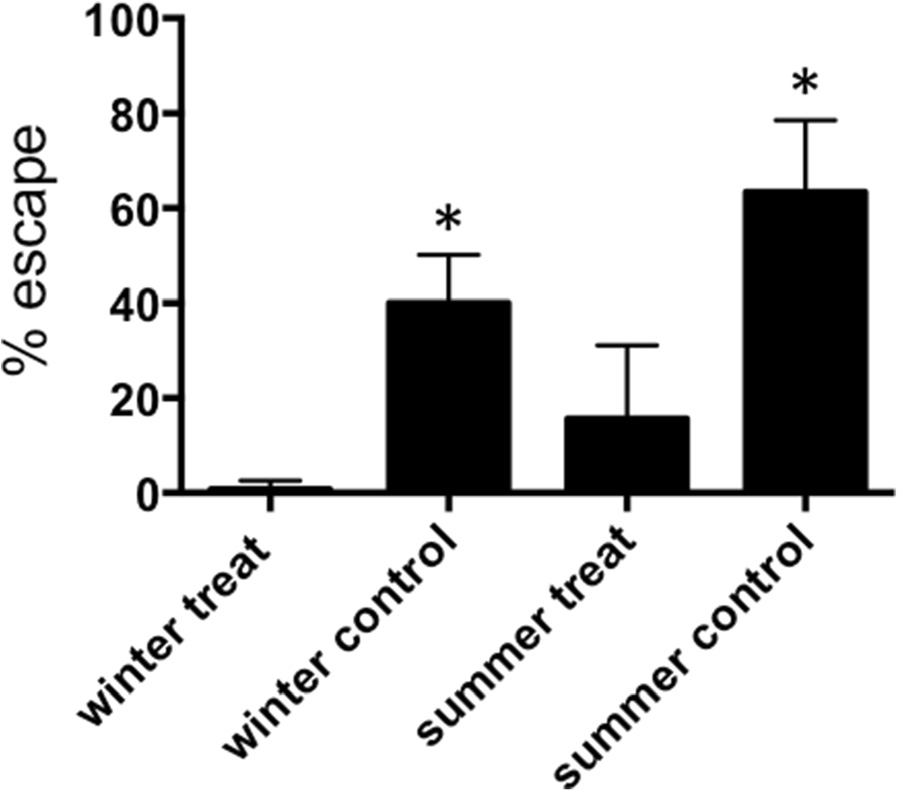
**Escape from metofluthrin treated rooms.** Mean (+ 95% CI) percent of released (30/cohort) female *Ae. aegypti* collected from window traps in a room containing a metofluthrin emanator. * depicts mean number captured between treatment vs. control that were significantly different (P < 0.05, paired t-test) within winter and summer trials.

In Room 1, under unexposed, controlled conditions, and in the absence of a human host, large numbers of female *Ae. aegypti* readily escaped into the window traps. In winter and summer respectively, the percentage of mosquitoes collected from traps after 24 hr, was 40 and 63% (Figure 3). In Room 2, in the absence of metofluthrin or human bait, the mean proportion collected in window traps after 20 minutes was 18.9% (CI: 10.8 – 27.0%). These were all live mosquitoes. These figures provided baselines for subsequent examination of the expellant impact of metofluthrin.

For the 5% formulation, in winter and summer, metofluthrin significantly reduced the mean number of females collected in window traps after 24 h (Figure 3). After 1 h, exposure, those few mosquitoes that were not KD flew slowly around the room, did not land or bite and often landed in harborage areas such as inside a laundry hamper or box. Dead mosquitoes were commonly noted on the floor, especially inside and near harborage areas.

**Figure 4:**
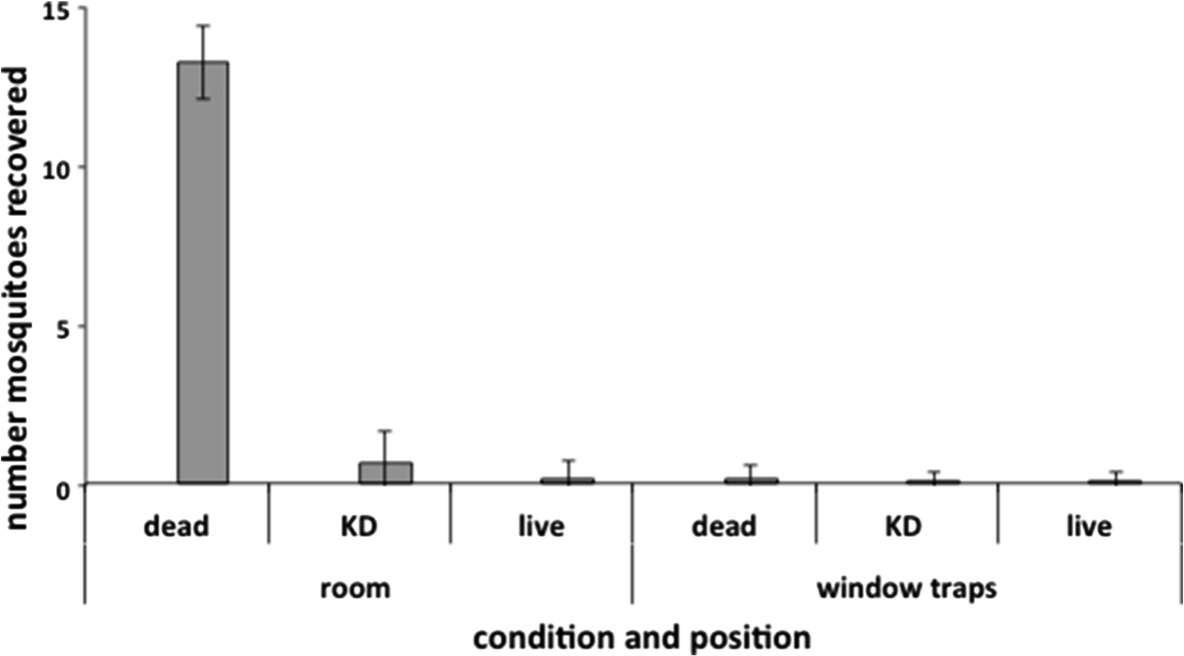
**Knockdown and escape with 10% AI metofluthrin.** Mean (CI) numbers of mosquitoes (from cohorts of 15) recovered from rooms after 20 minutes exposure.

In the presence of the 10% emanators, 90% of all host-seeking mosquitoes that were subsequently exposed to metofluthrin were dead after 20 minutes. The vast majority of mosquitoes dead, knocked down or alive, were recovered from the room (95%) mostly around harborage areas. Only 3.3% (CI 0–7.5%) of mosquitoes were found in the window trap. That is a significant reduction over the non-treated window trap catches (see above). Less than 2% of mosquitoes were unaccounted for. The means and 95% CIs for the condition and position of all mosquitoes collected after 20 minutes is given in Figure 4.

### Escape from open rooms

**Figure 5:**
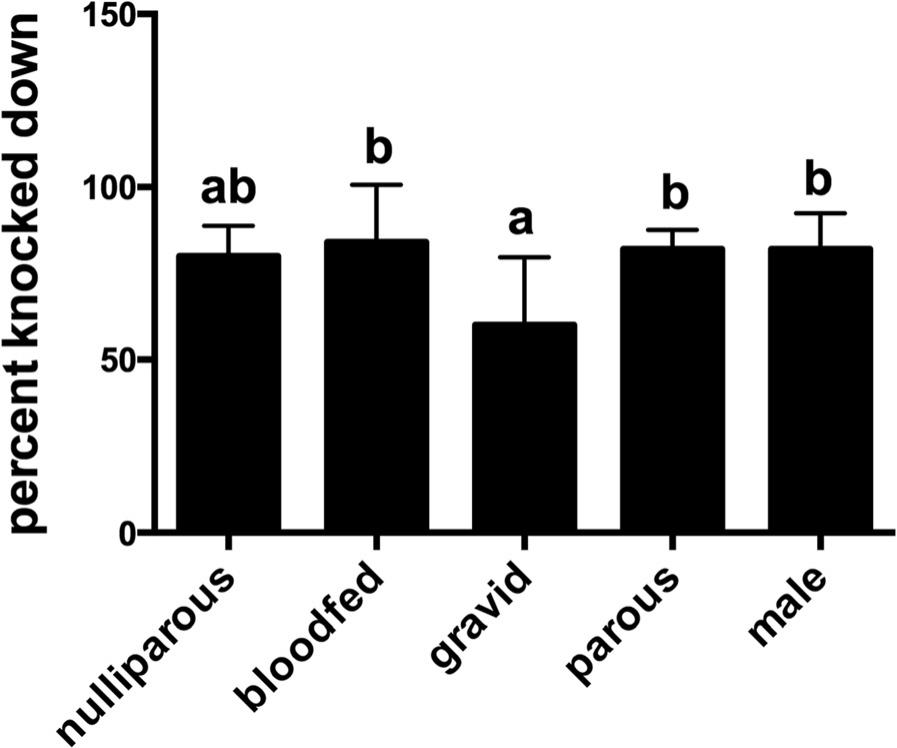
**Knockdown of *Aedes aegypti.*** Impact of metofluthrin on male and different physiological stages of female *Ae. aegypti*. The mean (CI) percent of mosquitoes (from cohorts of 10; *n =* 5) knocked down is presented, and letters depict significantly (P < 0.05) different means by Tukey’s multiple comparison test on log (X + 1) counts.

Both single and double doses of 5% resulted in significant (ANOVA, P < 0.001) KD and mortality of free-flying female *Ae. aegypti*. The mean (CI) KD for the untreated control, single and double 5% AI emanator treatments was 0 (0), 56% (4.9%) and 80% (6.3%), respectively. Furthermore, few mosquitoes escaped, with 30% leaving the room for the control, and ca. 28% and 13% escaping or not found for the single and double 5% emanators, respectively. The treatment and control escape numbers did not differ significantly (P = 0.07). Mortality of female *Ae. aegypti* in the sentinel cages with 0, single or double emanators after 1 h was 0%, 92% and 100%, respectively. Significant KD was observed in males and nulliparous, blood fed, gravid and parous female *Ae. aegypti* exposed to the double emanator for 2 hrs. KD of gravid females was significantly lower for gravid females (P < 0.05) than bloodfeds, parous and male *Ae. aegypti* (Tukey’s HSD) (Figure 5). The majority of exposed mosquitoes remained in the room. The mean (CI) percentage escaping/not found ranged from 16% (12%) (blood fed females) to 18% (8.0%/4.0%) (males/parous females) and 32% (15%) (gravid females).

### Behaviors of exposed *Ae. aegypti* individuals

**Figure 6:**
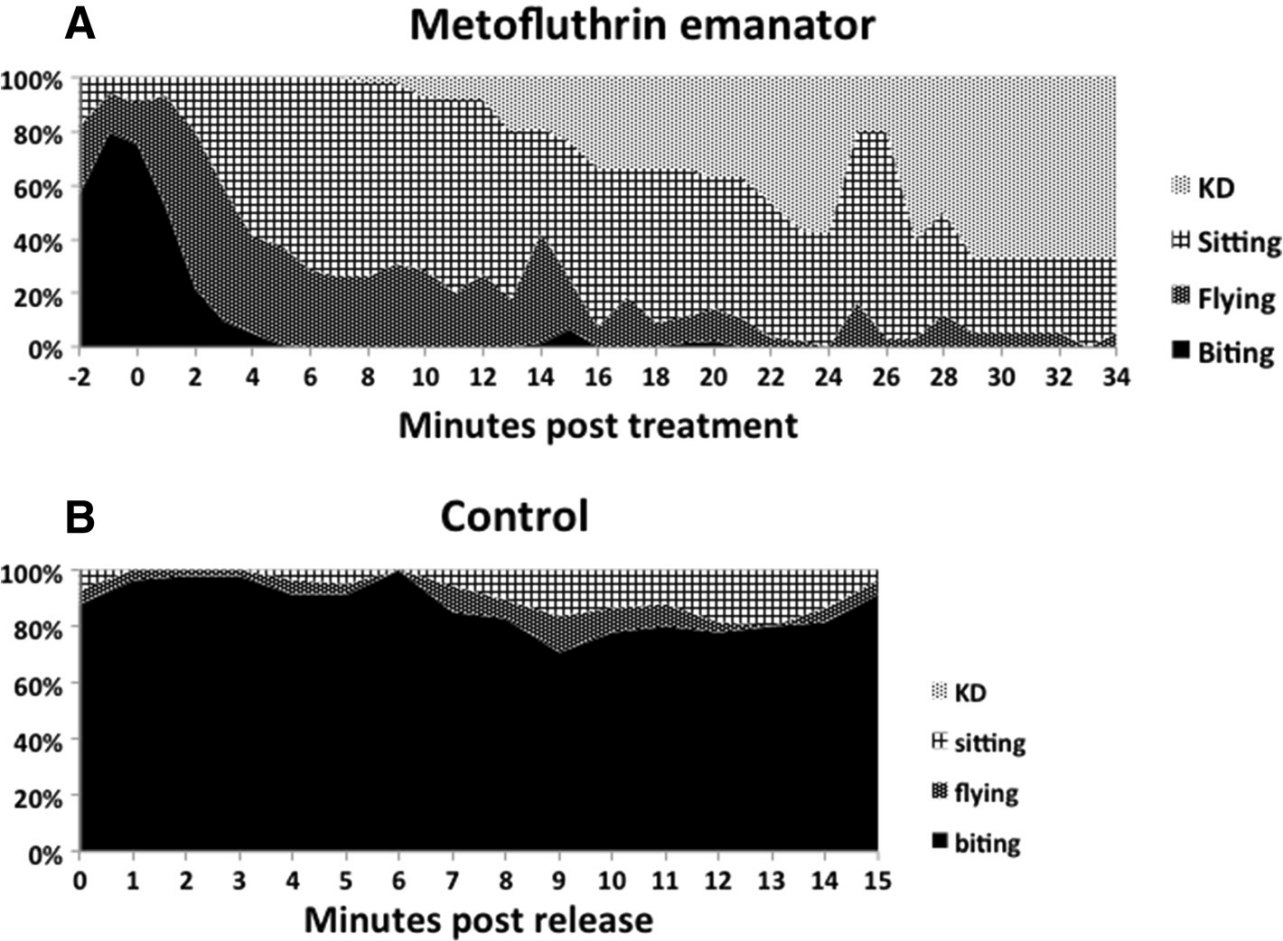
**Behavior of individual mosquitoes.** Pooled behavior of individual female *Ae. aegypti* exposed to the “double” 5% AI emanator **(A)** (n = 15) and **(B)** an untreated control (n = 10) in an open room.

Biting ensued soon after release of mosquitoes but within 2 minutes of exposure to a two 5% metofluthrin emanator, mosquitoes stopped landing on the host and began flying across the room from suitcase to suitcase. Biting decreased markedly and mosquitoes spent increasing periods resting on the harborage areas (Figure 6A).

Detailed observations showed that individual female *Ae. aegypti* slowly crawled up the face of the suitcases provided as harborage, before engaging in sporadic flights across the room. At 15–30 min., KD was noted, and usually occurred near the suitcase the female was resting on. Occasionally, a KD female would fly from the floor, but then was KD again within minutes. Of the 15 individual mosquitoes released, 5 (33%) escaped through the window, a rate comparable to the 30% escaping from the untreated control in the cohort study (see above). The observer could not discern any directed exit behavior and on two occasions a female exited only to fly back in within seconds. Untreated control mosquitoes bit throughout the 15-minute control period (Figure 6B).

### Studies on longevity of 10% metofluthrin emanators

**Figure 7:**
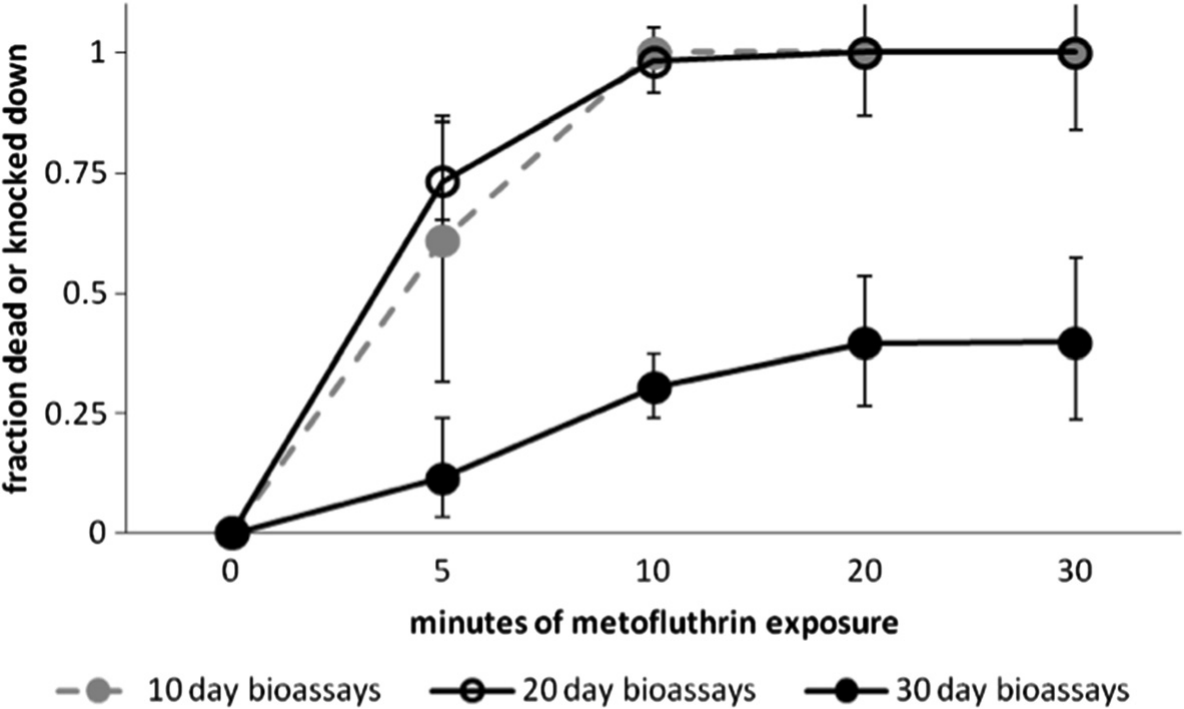
**Longevity of metofluthrin emanator.** Effect of 10% AI metofluthrin emanator age on KD and death of mosquitoes exposed in bioassay rings (mean ± 95% CI).

Bioassays using caged mosquitoes placed 1 m from the 10% emanators in Room 2 showed that the emanators lost efficacy once they had been open for 30 days: those aging emanators killed only 12% of mosquitoes after 20 minutes. In comparison, emanators that were 11 or 20 days old rendered almost all mosquitoes dead or KD in equal measure after 10 minutes and by 20 minutes 96% and 70% of mosquitoes were dead and the remainder were KD (repeated measures ANOVA; P < 0.01, F = 143). Mean effects and associated 95% confidence intervals are illustrated in Figure 7.

## Discussion

Metofluthrin has been in commercial use, in a variety of forms, for almost a decade [13]. More recently, its potential as a tool to prevent the transmission of disease has been considered [3,8] but with very few exceptions [3], tests of its impacts have focused on its impact at low doses. Mosquito coils containing 0.00625% w/w metofluthrin resulted in air concentrations of 0.02 - 0.06 μg/um^3^ and caused small reductions in entry rates and minimal KD or mortality in comparison to blank coils [4]. Ten-fold higher air concentrations (0.16 - 0.43 μg /m^3^) achieved 50% reductions in indoor resting densities [2]. The impact of entomological reductions at this scale on disease transmission are unknown [14].

In North Queensland, IRS is the most effective dengue control tool available. It kills indoor-resting viraemic mosquitoes and prevents infective bites [15]. Operationally however, it is laborious and expensive. The optimization of high dose metofluthrin emanators (and other volatile compounds) may result in fast, effective alternatives to IRS. That will require a clear determination of their impact under a range of environmental conditions (especially relating to room volumes, rates of air exchange and the biting behavior of mosquitoes exposed to sublethal doses). The effects that we have described here suggest a tremendous potential in terms of blocking disease transmission, especially for highly endophilic, anthropophagic disease vectors.

Impacts on free-flying mosquitoes will be dependent on dose, rate of volatilization and airflow. In large wind tunnels (64 m^3^), impregnated paper emanators (50 mg a.i / m^2^) positioned ca. 1 m from a human gave 90% reductions in biting rates within minutes of exposure [1]. No mention was made of mortality. At a slightly reduced dose (34 mg a.i / m^2^) 1 h of exposure was sufficient to prevent all biting in two connected rooms (totaling 50 m^3^). After 24 hours 98% of those exposed mosquitoes were dead. In both cases, the emanators were less than 3 days old.

New formulations of metofluthrin, in which even higher amounts of active ingredient are bound to polyethylene netting or to resins are being formulated. We have shown that these can give sustained release of operationally desirable doses over a 20–30 day period. When deployed in areas of active dengue transmission, that residual life would be sufficient to protect individual households from infection for very relevant periods of time (local mosquitoes carrying dengue infections are unlikely to survive for more than 20 days). When deployed at a community-wide level, the subsequent disruption to mosquito survival, resting behaviours and the ability to find a blood-meal may have additional impacts on overall mosquito numbers. In this respect it might be considered as a highly efficient, cost effective form of indoor residual spraying, in which endophilic, endophagic mosquitoes are exposed again and again, every time they seek a blood meal.

This study confirms that most female *Ae. aegypti* are not expelled from rooms treated with metofluthrin. This was confirmed by exit window trap collections, cohort studies in a room with open windows, and in observations of single females. In the latter, active flight ensued after exposure, and mosquitoes flew repeated across the face of open windows, but only 15-30% exited. They spent the majority of their time resting on harborage sites before being knocked down 15–30 min after exposure. With the 10% formulation, significant KD and mortality was seen after just 5 minutes among caged mosquitoes and free-flying mosquitoes began to stop biting immediately after exposure. Almost all were dead or KD after 20 minutes and very few exited the room. These observations on expellency may be important as escaping mosquitoes might survive, recover and engage in further host-seeking and disease transmission behaviors [10-12].

This suite of room studies may represent a best case scenario for metofluthrin. The rooms were small (< 25 m^3^), and there was no vigorous air exchange. Metofluthrin KD is expected to decrease significantly for larger rooms and in outdoor living areas such as verandas, patios and the spaces under high set homes (a common house type in North Queensland). Placement of the emanator near dark areas where *Ae. aegypti* might rest, or seating areas where human blood meals were often available could help target metofluthrin and improve control. That would be analogous to the use of targeted IRS in North Queensland where dark corners and the underside of furniture are most commonly treated.

This work indicates that metofluthrin acting as a fast and efficient alternative to IRS techniques, might be developed as a powerful dengue control tool. Activation of metofluthrin emanators indoors provided protection from biting, and killed mosquitoes. Comprehensive treatment of all indoor spaces in the vicinity of active disease transmission might provide rapid and thorough control of *Ae. aegypti*, and prevent infective bites. At least one of the formulations tested here (10% AI) worked effectively for operationally meaningful lengths of time.

Additional research is needed, however. We need to know: i) if mosquitoes resistant to synthetic pyrethroids remain affected by metofluthrin, ii) the limits of efficacy for emanators in larger spaces with greater airflow iii) the impacts of area-wide, operational use and iv) if trials in areas of active disease transmission can reduce the incidence of disease.

## Conclusion

The new, high-dose, resin formulations of metofluthrin act quickly to prevent biting and to knockdown and kill free-flying female *Ae. aegypti* in our experimental rooms. In room bioassays we have consistently shown that female *Ae. aegypti* are not expelled from rooms treated with a metofluthrin emanator despite the opportunity to fly through open windows. Observations of released females in rooms treated with the metofluthrin emanator indicate they rapidly cease biting, then fly throughout the room, before resting on harborage sites and eventual knockdown resulting in death. About 20-30% of the mosquitoes escaped the room, equivalent to the untreated control rate. We also demonstrated that the 10% AI emanator continued to knock down mosquitoes up to 20 days under field conditions. Resin-based metofluthrin emanators show great potential as a replacement for labor intensive IRS for dengue vector control.

## Competing interests

Scott Ritchie conducted part of this research that was funded by a grant from Sumitomo Chemical Australia to the Dr. Edward Koch Foundation, Cairns Australia. He received no salary from this grant.

## Authors’ contributions

SR designed and carried out room studies 1 and 3, behavioral assay and helped write the manuscript; GD designed and carried out room study 2, the emanator longevity study and helped write the paper. Both authors approved the final version of the manuscript.
